# Double-edge scan wavefront metrology and its application in crystal diffraction wavefront measurements

**DOI:** 10.1107/S1600577524006222

**Published:** 2024-07-29

**Authors:** Fang Liu, Ming Li, Qianshun Diao, Zhe Li, Zhibang Shen, Fan Li, Zhen Hong, Hongkai Lian, Shuaipeng Yue, Qingyan Hou, Changrui Zhang, Dongni Zhang, Congcong Li, Fugui Yang, Junliang Yang

**Affiliations:** a Beijing Synchrotron Radiation Facility, Institute of High Energy Physics Chinese Academy of Sciences Beijing100049 People’s Republic of China; b University of Chinese Academy of Sciences Chinese Academy of Sciences Beijing100049 People’s Republic of China; c National Institute of Metrology Beijing100029 People’s Republic of China; Tohoku University, Japan

**Keywords:** wavefront metrology, crystal diffraction, double-edge, diffraction-limited, wavefront error

## Abstract

Achieving diffraction-limited performance in fourth-generation synchrotron radiation sources demands monochromator crystals that can preserve the wavefront across an unprecedented extensive range. There is an urgent need for techniques of absolute crystal diffraction wavefront measurement. At the Beijing Synchrotron Radiation Facility (BSRF), a novel edge scan wavefront metrology technique has been developed. This technique employs a double-edge tracking method, making diffraction-limited level absolute crystal diffraction wavefront measurement a reality. The results demonstrate an equivalent diffraction surface slope error below 70 nrad (corresponding to a wavefront phase error of 4.57% λ) r.m.s. within a nearly 6 mm range for a flat crystal in the crystal surface coordinate. The double-edge structure contributes to exceptional measurement precision for slope error reproducibility, achieving levels below 15 nrad (phase error reproducibility < λ/100) even at a first-generation synchrotron radiation source. Currently, the measurement termed double-edge scan (DES) has already been regarded as a critical feedback mechanism in the fabrication of next-generation crystals.

## Abstract 2

An innovative double-edge scan wavefront metrology method has been developed which can resolve the problem of high-precision crystal absolute diffraction wavefront measurements. This method can be regarded as a critical feedback mechanism in the processing of next-generation crystals.

## 1. Introduction

Crystal monochromators serve as crucial optical components in hard X-ray beamlines, finding applications in diffraction, imaging and spectroscopy experiments (Beckhoff *et al.*, 2007 ▸; Okamura *et al.*, 2010 ▸; Bergeard *et al.*, 2011 ▸; Pankratov & Kotlov, 2020 ▸; Chan *et al.*, 2020 ▸). The fourth-generation synchrotron radiation (4th GSR) sources boast a transverse coherence theoretically two orders of magnitude larger than that of their third-generation counterparts (3rd GSR). To fully utilize the diffraction-limited capabilities of 4th GSR sources, crystals must preserve wavefronts over a significantly more extensive range, with the r.m.s. diffraction wavefront phase error needing to be within λ/14 according to the Maréchal criterion (Maréchal, 1947 ▸). Consequently, the ability to preserve wavefronts becomes a new standard for evaluating crystal quality.

Over the decades, various crystal fabrication techniques, including traditional chemical–mechanical polishing (Khachatryan *et al.*, 2004 ▸; Kasman *et al.*, 2015 ▸, 2017 ▸), damage-free polishing without chemical additives (Biddut *et al.*, 2008 ▸), state-of-the-art plasma chemical vaporization machining (Hirano *et al.*, 2016 ▸; Katayama *et al.*, 2019 ▸), magnetically controlled chemical–mechanical polishing (Hong *et al.*, 2023 ▸) and others, have been developed. The fabrication of high-quality crystals has become a reality. However, the technology for measuring crystal Bragg diffraction wavefronts is still underdeveloped. Because of diffraction geometry, a pure relative measurement comparing wavefronts with and without the crystal is challenging due to beam flipping and image blurring from the crystal extinction effect with an extinction length typically ∼1–100 µm, depending on the Bragg diffraction conditions (Cocco *et al.*, 2022 ▸; Shi *et al.*, 2023 ▸). A possible approach involves using a speckle scanning technique measuring a double-crystal setup providing a parallel exit beam relative to the incident beam, as demonstrated for channel-cut crystals with much larger phase errors (Xue *et al.*, 2020 ▸). Recently, a method based on a coded mask realized phase error sensitivity at the λ/100 level, but only for the self-referencing mode (relative wavefront error in different crystal areas), and could achieve absolute wavefront measurement for double crystals only (Shi *et al.*, 2023 ▸). The absolute diffraction wavefront measurement can reveal wavefront distortion caused by crystals more directly. However, this method still involves uncertainties in the absolute mode when tracking between directed and blurred diffraction beam images.

Considering the Hartmann method typically characterizes relatively low spatial frequencies (Rochefoucauld *et al.*, 2021 ▸), the pencil-beam method is a possible solution for measuring crystal diffraction wavefronts, since it minimally affects measurement accuracy in image blurring and is capable of delivering shape errors with much higher spatial frequencies. As early as 1997, the pencil-beam method was used as an X-ray long trace profiler to measure surface slope errors for a mechanical bending mirror (Hignette *et al.*, 1997 ▸). Precision and accuracy can be better than 25 nrad r.m.s. and 50 nrad r.m.s., respectively, although with poor lateral resolution of 5 mm in the meridional direction and about 1 mm in the sagittal direction. The high precision mainly resulted from the long distance between the mirror and detector (1.85 m), requiring considerable space. In recent years, the *in situ* pencil-beam method has been utilized at many synchrotron radiation sources to measure the slope error of Kirkpatrick–Baez mirrors, X-ray active deformable mirrors and to provide feedback for optimizing focusing parameters of deformable mirrors for various experiment requirements (Yuan *et al.*, 2010 ▸; Merthe *et al.*, 2011 ▸; Sutter *et al.*, 2012 ▸, 2014 ▸; Goto *et al.*, 2016*a* ▸). As reported by Sutter, the centroid calculation is reproducible to within only 0.1 pixels (Sutter *et al.*, 2012 ▸). Similarly, Nakamori *et al.* used the pencil-beam method to correct a piezoelectric deformable mirror and realize nanofocusing but the slope measurement resolution was only expected to be of the order of 1 µrad (Nakamori *et al.*, 2013 ▸). Goto *et al.* investigated the accuracy of the pencil-beam method. They realized reproducibility of ∼40 nrad with a specially developed high-magnification X-ray beam monitor but for a focusing mirror measurement of only 38 mm in length at SPring-8, a 3rd GSR source (Goto *et al.*, 2016*b* ▸). However, the traditional pencil-beam method still faces challenges, including ultrahigh stability requirements.

In this work, we describe an innovative wavefront metrology method developed at the Beijing Synchrotron Radiation Facility (BSRF) named the double-edge scan (DES) wavefront metrology technique to characterize the absolute crystal diffraction wavefront in one direction. Firstly, we provide detailed instructions on the principles and advantages of the DES method. Secondly, we conduct diffraction wavefront measurements of a high-quality flat crystal and a channel-cut crystal. Thirdly, we carry out a reproducibility comparison between double-edge mode and single-edge mode to demonstrate the outstanding advantages of the double-edge structure. Finally, we perform a shape error comparison measurement of an X-ray flat mirror using the DES method and a self-developed long trace profiler (LTP).

## 2. Methods

**Figure 1:**
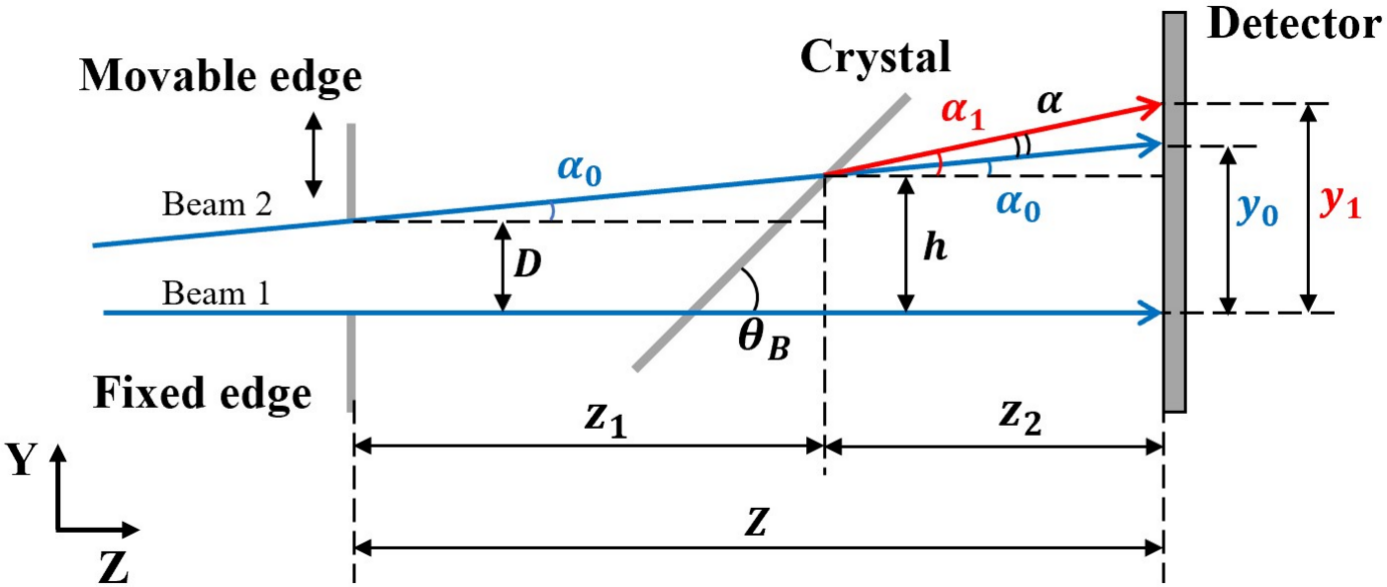
Equivalent X-ray beam geometry for the DES method when measuring a crystal sample.

As the Bragg diffraction angle equals the incident angle, Fig. 1 ▸ illustrates the equivalent X-ray beam geometry. The crystal sample diffracts a beam passing through the double-edge structure (comprising a movable edge and a fixed edge) at the Bragg diffraction angle θ_B_, which then is received by the detector. Specifically, we label the beam passing through the fixed edge as beam 1 and the one passing through the movable edge as beam 2. The distance between the two edges along the *Y* direction is *D*. Initially, we remove the crystal sample from the beam path. Subsequently, we record projections of both the movable and fixed edges using the detector.

The quantity *y*_0_ represents the distance between the two edge projections on the detector. *Z* denotes the distance between the double-edge structure and the detector along the *Z* direction. Consequently, we express the incident reference wavefront slope α_0_ as
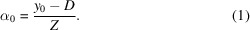
The distance *D* can be precisely measured during the scanning process with nanometre precision using a laser interferometer. Thus, the precision of α_0_ measurement relies on the laser interferometer instead of the positioning accuracy of the scanning stage. Subsequently, with the crystal sample introduced into the beam path and considering the diffraction wavefront error introduced by the crystal, beam 1 serves as the reference. Beam 2 then propagates along the direction of α_1_, and the detector records the distance *y*_1_ between the two edge projections. The slope α_1_ is
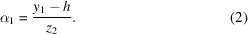
Here, we define the plane where beam 2 reaches the crystal sample and is perpendicular to the *Z* axis as the crystal plane. The parameter *h* represents the distance between the two beams along the *Y* direction in the crystal plane. The parameters *z*_1_ and *z*_2_ denote the distance from the double-edge structure to the crystal plane and from the crystal plane to the detector along the *Z* direction, respectively. We can derive the parameters (*h*, *z*_1_ and *z*_2_) from simple geometric relationships.

As Bragg diffraction is analogous to mirror reflection, we define the equivalent diffraction surface (EDS) slope error α to depict the absolute crystal diffraction wavefront slope error. Thus, α is
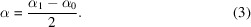


In the DES method, edge projections are used to track beams. The image blurring caused by the crystal extinction effect (Shi *et al.*, 2023 ▸) merely broadens the edge projections, ensuring minimal impact on the positioning accuracy of a single edge projection. This condition is paramount for achieving high-precision crystal diffraction wavefront measurements. The fixed edge is a reliable reference during beam flipping when measuring a flat crystal. When combined with a laser interferometer, this approach enables high-precision absolute wavefront measurements for single flat crystals, also including channel-cut crystals. The DES method is instrumental in addressing critical challenges associated with the first-generation synchrotron radiation (1st GSR) source, which is pivotal in achieving high-precision measurements. As the DES is not contingent on the transverse coherence of sources to generate clear modulation patterns, beam tracking and positioning accuracy remain unaffected by the transverse coherence of sources. In contrast to the traditional pencil-beam method, which relies on absolute beam position measurements (such as centroid positioning) susceptible to beam direction instability (drift of the source and sample position, tilt of the reference wavefront), the DES method employs a double-edge structure. By employing this structure, we measure the relative distance *y*_0_ (or *y*_1_), which remains largely unaffected. Thus, we can deduce the influence of beam direction instability and perform high-precision measurements. For curved wavefront measurement, the DES method has greater advantages in large-curvature wavefront characterization without the problem of tracking failure.

In the DES method, the angular resolution is limited by Δ*y*/*z*_2_, where Δ*y* is the minimum detectable edge displacement distance on the detector and *z*_2_ is the distance between the sample and detector. Both increasing the distance *z*_2_ and decreasing the distance Δ*y* can improve the angular resolution. The distance Δ*y* will be mainly limited by the raw data signal-to-noise ratio, the centroid positioning algorithm, the image resolution of the detector and the stability of the experiment system. The spatial resolution of the DES method will be limited by the width of the first diffraction zone (λ*Z*)^1/2^ (Lang *et al.*, 2014 ▸), where λ is wavelength, *Z* is the distance between the edges and detector.

## 3. Experimental setup

**Figure 2:**
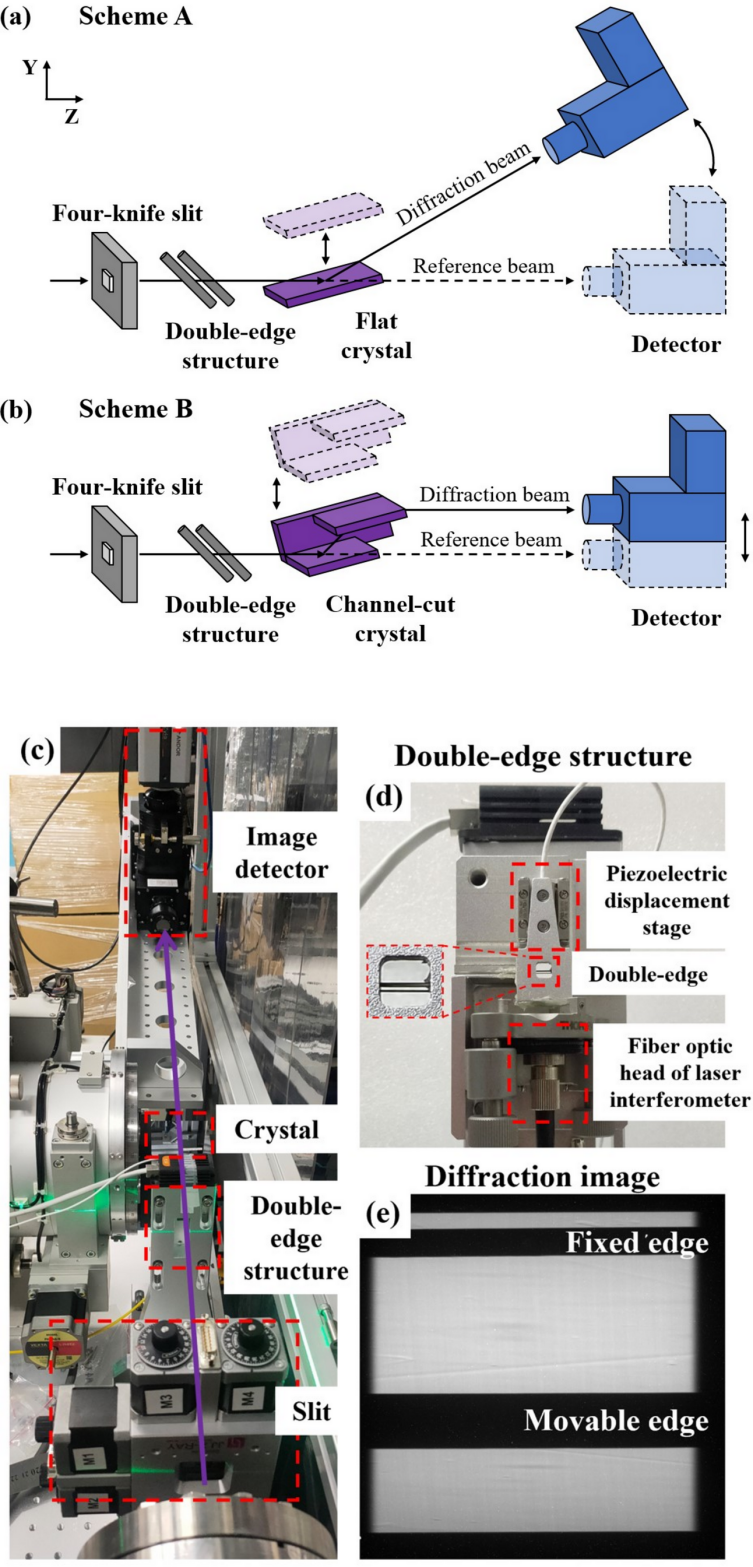
Schemes A and B depict the measurement of wavefront distortions during diffraction from a flat crystal sample (*a*) and a channel-cut crystal sample (*b*), respectively. The dashed lines illustrate the reference beam path when the crystal(s) and detector move to the dotted positions. Photograph (*c*) visualizes the DES system, while photograph (*d*) showcases the double-edge structure. The diffracted image (*e*) captures the result with a flat crystal sample in the beam path.

We conducted the experiment at the 1B3B beamline at the BSRF, employing X-rays monochromated by a Si(111) channel-cut monochromator positioned 23.5 m downstream of the bending-magnet source. The X-ray energy we used was 15 keV with about 4 eV energy bandwidth. Fig. 2 ▸ illustrates the DES wavefront characterization setup positioned approximately 26 m downstream of the source. A four-knife slit was utilized to restrict beam size, with the four knife projections visible at the detector for correcting misalignment caused by the miscut angle between images with and without the crystals. Positioned upstream of the crystal, the double-edge structure consists of a fixed edge and a movable edge, with the edges required to be smooth without obvious defects, and the material to be of low penetrability for the energy employed. Actually, the edge profile is fully consistent from one step to the next, so the measurement is largely unaffected by the edge surface quality. We used tungsten wire in this setup, placed as close as possible (about 10 cm) to the crystal sample to improve spatial resolution. The imaging detector, consisting of a 50 µm-thick LuAG:Ce scintillator, a 4× objective lens (NA = 0.16) and an Andor-Zyla-4.2P camera (6.5 µm per pixel), achieved an imaging resolution better than 3 µm. We positioned the detector approximately 0.48 m away from the sample center. A SmarAct-SLC-1720 piezoelectric displacement stage was the scanning stage, with the movable edge fixed. The laser interferometer used was the attocube IDS3010. We used a bicircular diffractometer to adjust the crystal Bragg diffraction angle θ_B_ and the detector tilt angle 2θ_B_. To enhance system stability, the four-knife slit, double-edge structure and bicircular diffractometer were mounted on the same aluminium base plate.

In the experiment, the fixed edge remained stationary, while the movable edge scanned along the *Y* direction. In order to improve the accuracy and reduce sensitivity to linear slow drift, the movable edge went back and forth for one measurement (Yashchuk, 2009 ▸). Photos were taken to record the distance *y*_0_ or *y*_1_ and the distance *D* was measured by the laser interferometer at each scan step. It needs 20 s for a single exposure because of the low X-ray flux from the 1st GSR bending magnet. We measured flat and channel-cut crystals with different geometries, as depicted in Figs. 2 ▸(*a*) and 2 ▸(*b*). By scanning the movable edge along the *Y* direction, the deflection angle can be directly measured without and with a crystal sample according to equations (1) and (2). Then the absolute crystal diffraction wavefront slope can be calculated via equation (3). The main steps for a data analysis program are fitting the edge shadow intensity distribution with an error function and calculating the centric position of the fitting function to obtain the position of the edge shadow.

## 4. Experimental results

### 4.1. Measurement 1: fine scanning measurement

**Figure 3:**
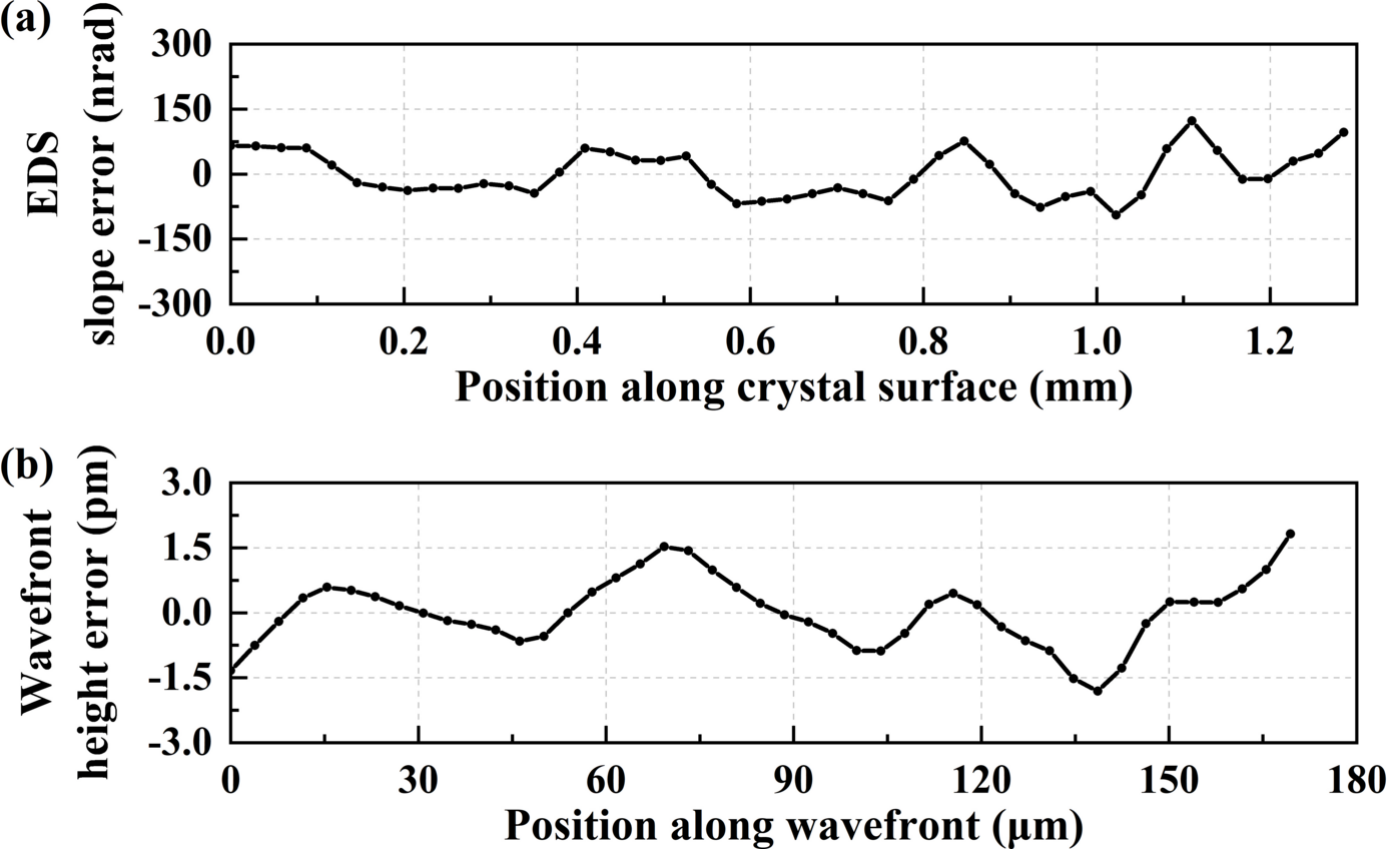
(*a*) Crystal EDS slope error profile in fine scanning. (*b*) Wavefront height error profile in fine scanning.

Firstly, we conducted a fine scanning measurement with a self-fabricated high-quality flat Si(111) crystal using scheme A, as demonstrated in Fig. 2 ▸(*a*). The step size (Δ*d*) was 3.85 µm in the wavefront coordinate and approximately 29 µm (Δ*l*) in the crystal surface coordinate with a linear relationship Δ*d* = Δ*l*sinθ_B_, 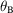 = 7.57°. In the data processing, the raw slope data were linearly fitted and a uniform tilt value was subtracted to obtain slope error (same in the later data processing). As the spatial resolution is limited by (λ*Z*)^1/2^, the spatial resolution is therefore about 7 µm in the wavefront coordinate for the current experimental conditions which corresponds to approximately 53 µm in the crystal surface coordinate in the meridional direction. Thus, we chose a close integration width (about 50 µm) in the sagittal direction (same in the later data processing). The crystal EDS slope error profile is depicted in Fig. 3 ▸(*a*), while the absolute crystal diffraction wavefront height error, calculated through integration of slope error, is presented in Fig. 3 ▸(*b*).

The crystal EDS slope error measures 51.99 nrad r.m.s., and the wavefront height error is 0.79 pm (0.95% λ) r.m.s., achieving the λ/100 level. The r.m.s. phase error corresponds to a Strehl ratio (SR) of 0.996. The calculation of SR employs the equation
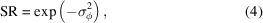
where 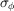 represents the r.m.s. phase error over the measurement range.

As shown in Fig. 3 ▸(*b*), the dominant spatial frequency of wavefront height error is about 50 µm in the wavefront coordinate. Therefore, the step size needs to be within 25 µm. Considering the measurement efficiency, we chose a step size of about 18 µm in later crystal measurements.

### 4.2. Measurement 2: flat crystal diffraction wavefront measurement

**Figure 4:**
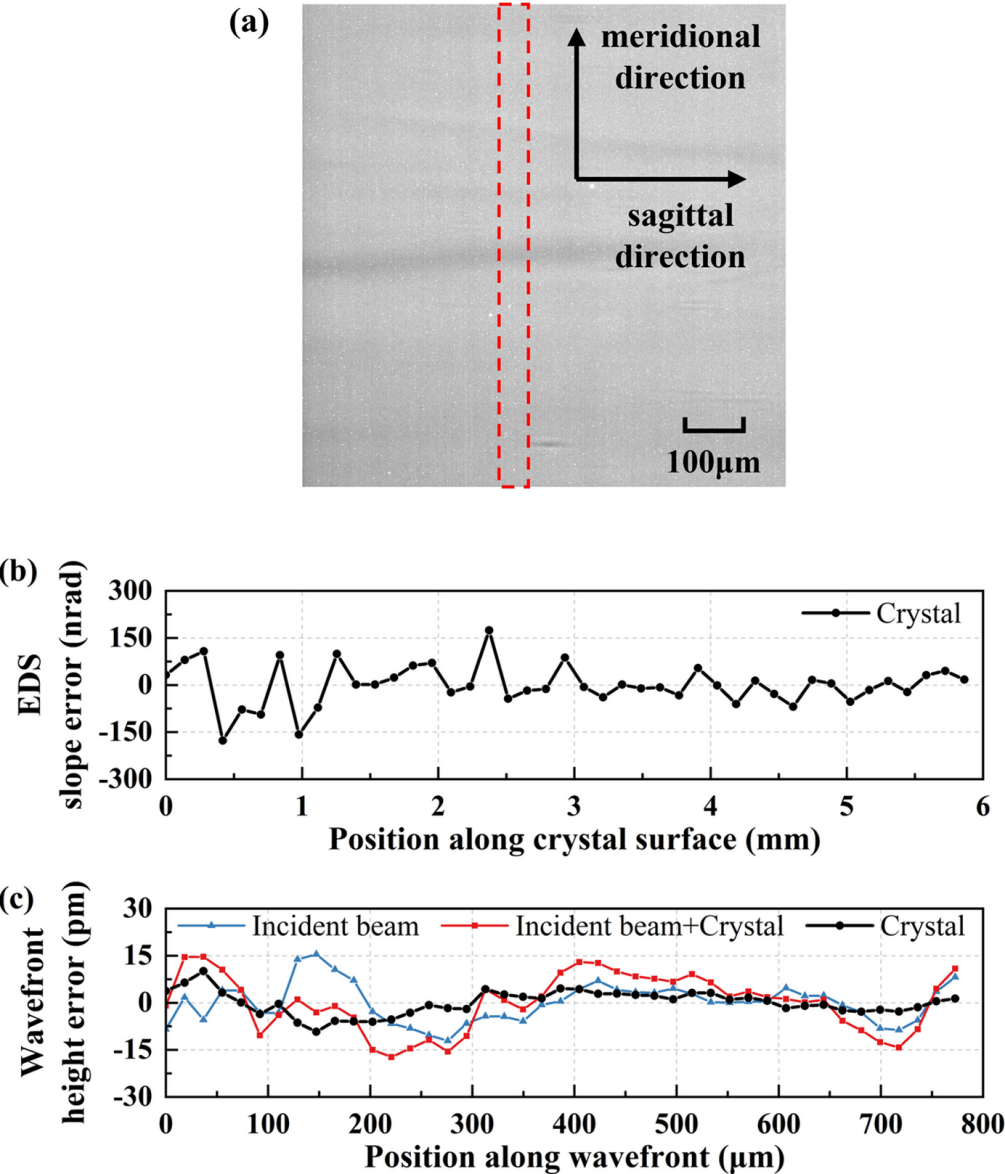
(*a*) X-ray diffraction image capturing the self-fabricated high-quality flat crystal. (*b*) Profile of crystal EDS slope error within the red dashed frame along the meridional direction in (*a*). (*c*) Profiles of incident wavefront height error without a crystal sample, with a crystal sample and absolute crystal diffraction wavefront height error.

Secondly, we measured the diffraction wavefront of a self-fabricated high-quality flat Si(111) crystal in a range of about 6 mm in the crystal surface coordinate. We captured an X-ray diffraction image for the flat crystal, as presented in Fig. 4 ▸(*a*). The image exhibits nonuniformity, with minor flaws originating from the crystal and stripes cutting across the entire image stemming from the incident reference X-ray beam. In this experiment, the scanning step size (Δ*d*) was 18.4 µm in the wavefront coordinate.

As mentioned in measurement 1, we selected approximately 50 µm in the sagittal direction for integration, as indicated by the red dashed frame in Fig. 4 ▸(*a*). Figs. 4 ▸(*b*) and 4 ▸(*c*) depict the crystal EDS slope error profile and the absolute crystal diffraction wavefront height error profile along the meridional direction, respectively. As the absolute crystal diffraction wavefront can be derived from the direct incident wavefront measurement with and without a crystal sample following equation (3), the incident wavefront height error profiles with and without a crystal sample are also depicted in Fig. 4 ▸(*c*).

The crystal EDS slope error measures 65.91 nrad r.m.s., and the absolute crystal diffraction wavefront height error is 3.78 pm (4.57%   λ) r.m.s., corresponding to a SR of 0.92. The outstanding results indicate that the crystal meets the wavefront preservation requirements of diffraction-limited fourth-generation X-ray sources, where according to the Maréchal criterion (Maréchal, 1947 ▸) the r.m.s. wavefront error must be below λ/14.

### 4.3. Measurement 3: channel-cut crystal diffraction wavefront measurement

**Figure 5:**
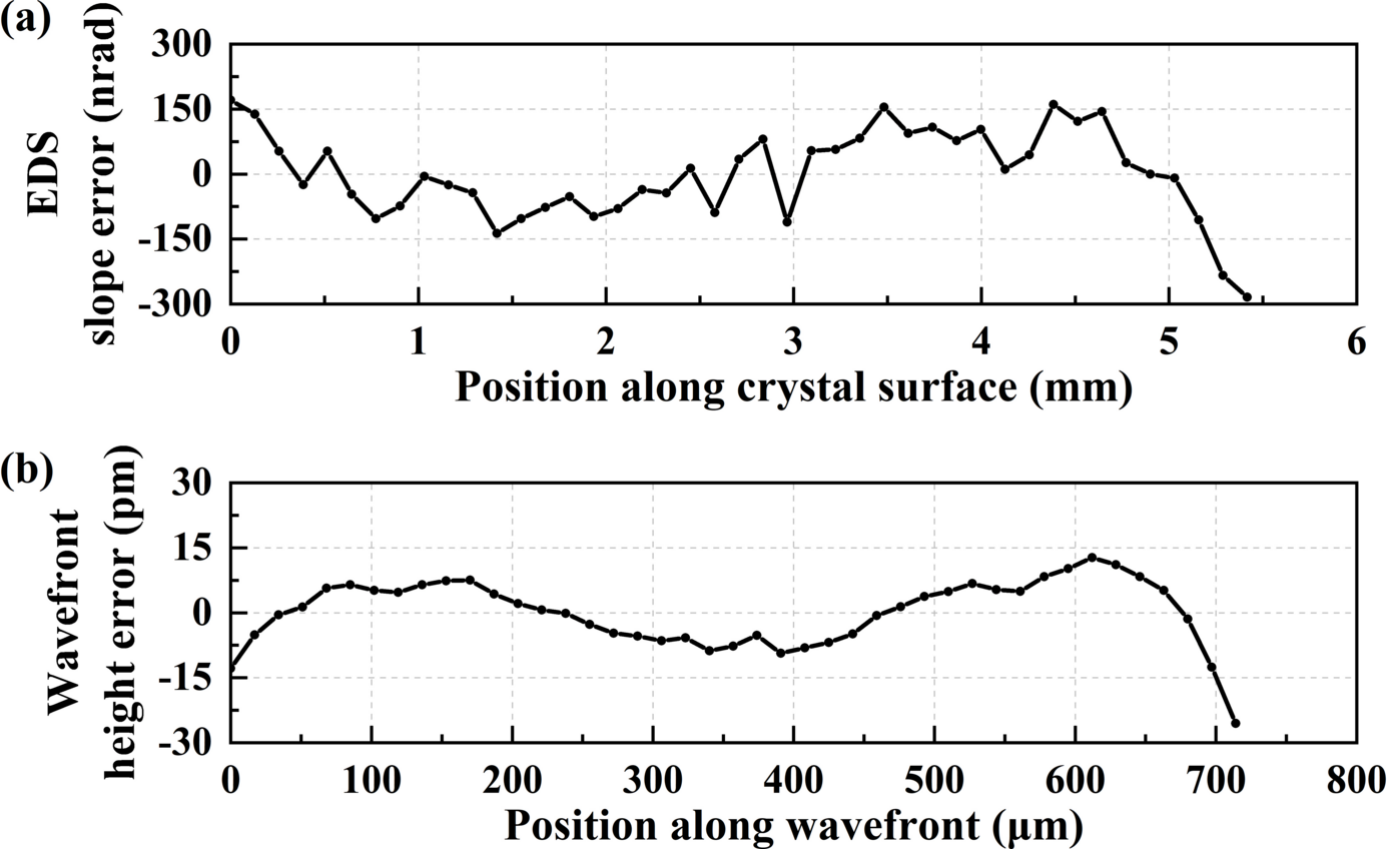
(*a*) Crystal EDS slope error profile of a channel-cut crystal. (*b*) Wavefront height error profile of a channel-cut crystal.

Thirdly, we measured a self-fabricated channel-cut Si(111) crystal in a range of about 5.5 mm in the crystal surface coordinate. The scanning step size (Δ*d*) was 17 µm in the wavefront coordinate. Figs. 5 ▸(*a*) and 5 ▸(*b*) depict the crystal EDS slope error profile and the absolute crystal diffraction wavefront height error profile, respectively. The crystal EDS slope error is 101.73 nrad r.m.s., and the wavefront height error is 7.65 pm (9.25% λ) r.m.s. As the fabrication of a channel-cut crystal is much more difficult because of the narrow gap between the two crystal surfaces, the wavefront error is slightly worse than 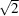 times that of the flat crystal.

### 4.4. Measurement 4: reproducibility measurement

Fourthly, to demonstrate the crucial role of the double-edge structure in achieving high-precision measurements, we compared the double-edge and single-edge modes. We performed a reproducibility experiment using a commercial flat Si(111) crystal from a Japanese company (EXCEED), involving three measurements with a scanning step size Δ*d* of 15 µm in the wavefront coordinate.

**Figure 6:**
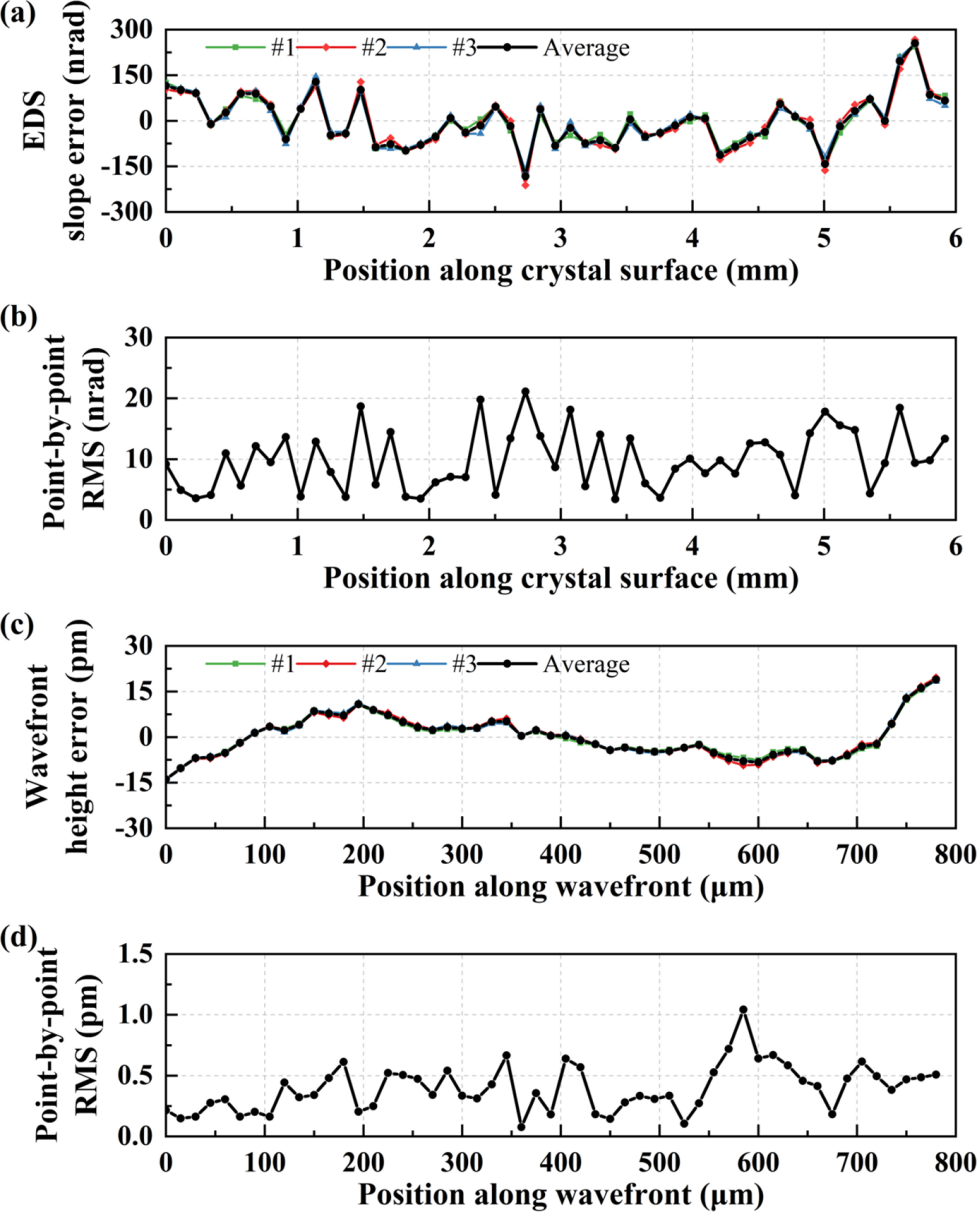
(*a*) Crystal EDS slope error profiles for three measurements and the average profile. (*b*) Point-by-point r.m.s. curve after subtracting the average slope error from three measurements. (*c*) Wavefront height error profiles for three measurements and the average profile. (*d*) Point-by-point r.m.s. curve after subtracting the average height error from three measurements.

In the double-edge mode, Fig. 6 ▸(*a*) illustrates the measured crystal EDS slope error profile for each measurement and the average slope error profile. Fig. 6 ▸(*b*) displays the three-point r.m.s. profile after subtracting the average slope error from three measurements. The average slope error is 82.08 nrad r.m.s., and the measurement-to-measurement reproducibility is 13.51 nrad. The precision was mainly limited by the signal-to-noise ratio of the raw data, the imaging resolution, changes in the shape of the incident wavefront between measurements with and without a sample. The wavefront height error profiles and the three-point r.m.s. profile after subtracting the average height error from three measurements are presented in Figs. 6 ▸(*c*) and 6 ▸(*d*), respectively. The average wavefront height error is 6.71 pm (8.11% λ) r.m.s., and the reproducibility is 0.54 pm (0.65% λ) which is below λ/100.

Next, we used the same raw data to reconstruct the wavefront for the single-edge mode to compare the measurement results with the double-edge mode. In the single-edge mode, instead of calculating the relative distance *y*_0_ or *y*_1_ for every scanning step, we focused on determining the absolute position of the movable edge.

**Figure 7:**
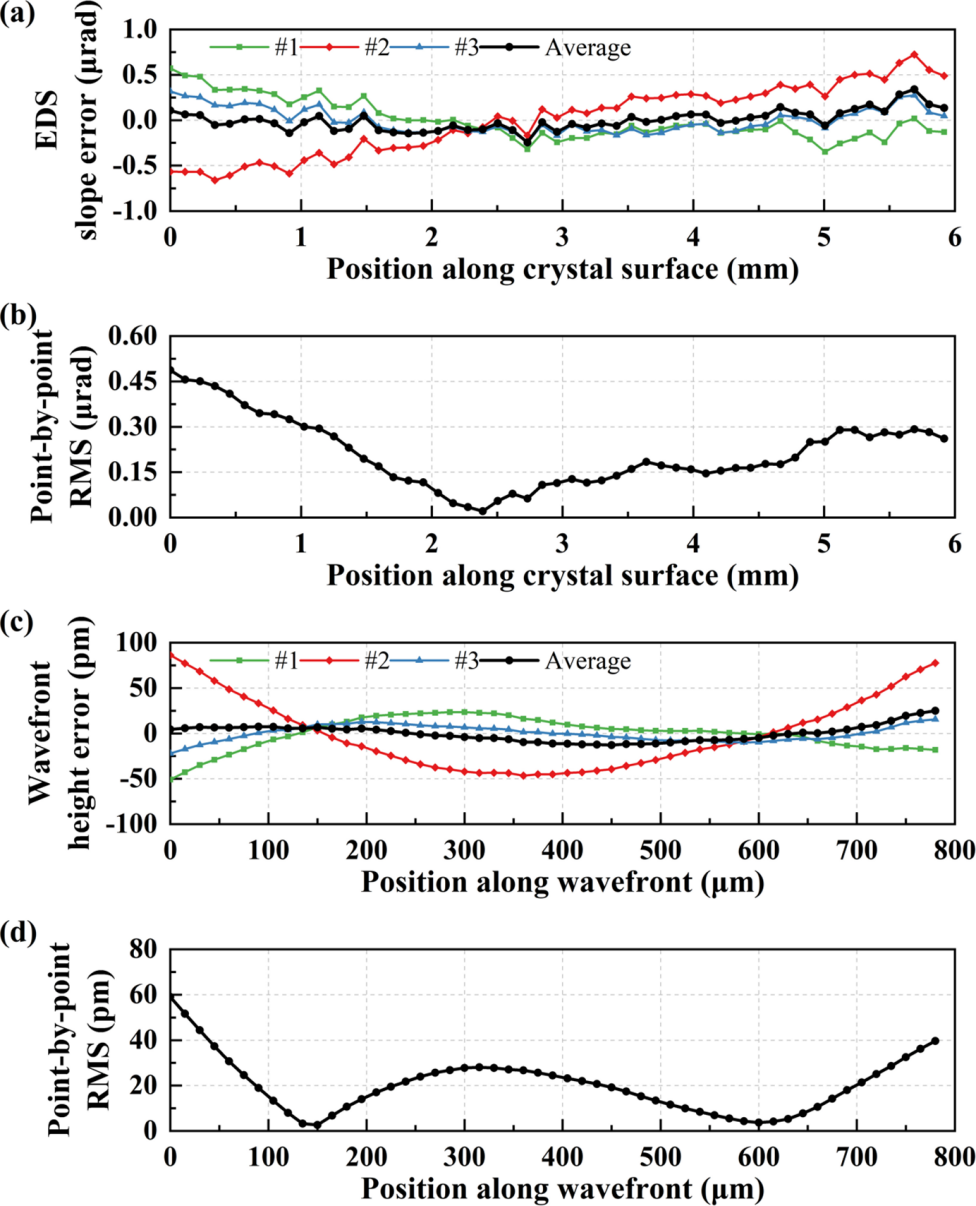
(*a*) Crystal EDS slope error profiles of three measurements and average profile. (*b*) Point-by-point r.m.s. curve after subtracting the average slope error from three measurements. (*c*) Wavefront height error profiles of three measurements and average profile. (*d*) Point-by-point r.m.s. curve after subtracting the average height error from three measurements.

Fig. 7 ▸(*a*) displays the reconstructed slope error profile for each measurement and the average slope error profile. Fig. 7 ▸(*b*) illustrates the three-point r.m.s. profile after subtracting the average slope error from three measurements. Additionally, Figs. 7 ▸(*c*) and 7 ▸(*d*) present the wavefront height error profiles and the three-point r.m.s. profile after subtracting the average height error from three measurements, respectively. The average slope error measures 109.99 nrad r.m.s., with a reproducibility of 296.33 nrad. The average wavefront height error is 8.94 pm (10.81% λ) r.m.s., and the reproducibility is 28.99 pm (35.05% λ). The reproducibility is nearly three times worse for the single-edge mode, potentially attributable to the nonlinear directivity slow drift of the incident beam, for it needs about an hour for one measurement. This comparison underscores the significant importance of the double-edge structure in reducing repetitive measurement errors and enhancing measurement reliability.

### 4.5. Measurement 5: comparative measurement with LTP

In the latest experiment, we conducted a comparative shape error measurement of a flat mirror using both the DES method and LTP. The mirror, coated with tungsten, measured 200 mm long with a 177 mm effective length. The uniform scanning step size was 0.8 mm on the mirror surface coordinate for the DES method and LTP.

**Figure 8:**
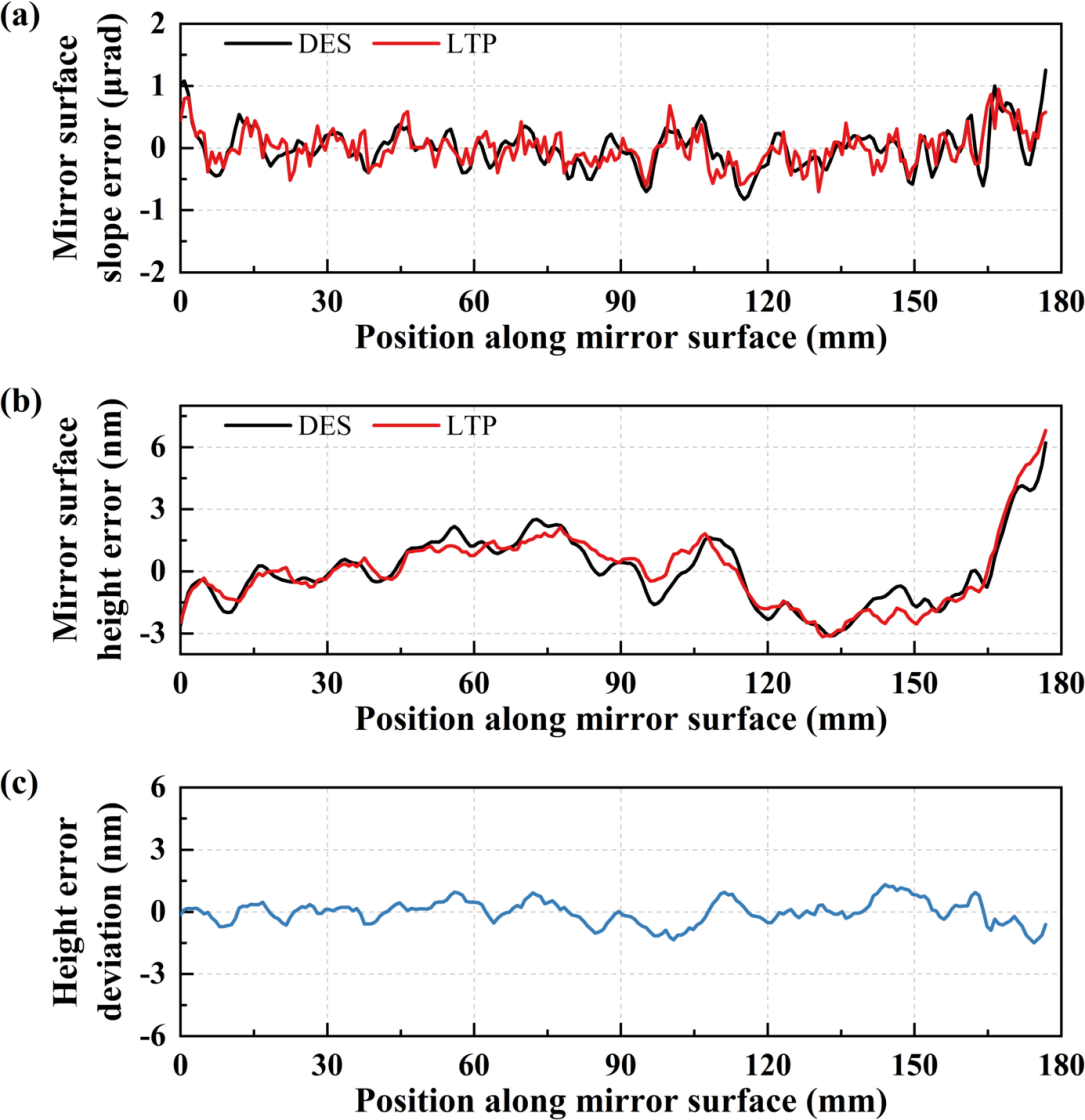
(*a*) Mirror surface slope error measured using the DES method and LTP. (*b*) Mirror surface height error profiles. (*c*) Surface height error deviations between the two methods.

Fig. 8 ▸(*a*) presents both methods’ measured surface slope error profiles. The slope error measures 0.294 µrad r.m.s. for the DES method and 0.344 µrad r.m.s. for the LTP. Subsequently, the slope errors were integrated into height errors, as depicted in Fig. 8 ▸(*b*). Notably, the surface height error profiles exhibit high similarity, with r.m.s. height errors of 1.69 nm for the DES method and 1.76 nm for the LTP. Fig. 8 ▸(*c*) illustrates the deviation in surface height errors between the two methods, indicating an r.m.s. of 0.57 nm. While nuanced differences in height errors between the two methods may stem from difference in spatial resolution and misalignment of the start position for scanning, the overall strong agreement in the mirror shape error between the LTP and the DES method represents a significant advancement. It lends robust support to the DES method.

## 5. Conclusions

We introduced the DES wavefront metrology technique at the BSRF, providing a groundbreaking solution for characterizing absolute diffraction wavefronts in high-quality monochromator crystals essential for diffraction-limited 4th GSR sources. The DES method, overcoming limitations in transverse coherence, beam direction instability and incident wavefront distortion, successfully achieved diffraction-limited level wavefront metrology on the 1st GSR source.

Our measurements demonstrated impressive results for a flat crystal, with a crystal EDS slope error of 65.91 nrad r.m.s. and a wavefront height error of 3.78 pm (4.57% λ) r.m.s. over a nearly 6 mm range. The crystal EDS slope error measured 101.73 nrad r.m.s. for a channel-cut crystal, and the wavefront height error was 7.65 pm (9.25% λ) r.m.s. over a nearly 5.5 mm range. The double-edge structure design has significant advantages in improving measurement precision, for we have achieved a wavefront slope error measurement reproducibility below 15 nrad (phase error reproducibility < λ/100), which meets the requirements for characterizing the diffraction wavefront of high-quality crystals. In a comparative measurement of a mirror using both the DES method and LTP, we determined a quantitative agreement for the surface height error (1.69 nm and 1.76 nm r.m.s., respectively), and similar prominent features were noted for the surface slope error profiles, providing robust support for the DES method.

Already recognized as crucial feedback in next-generation crystal fabrication, the DES method holds promise even for the 1st GSR source and could exhibit superior performance in more advanced synchrotron radiation sources. The DES method will be pivotal in X-ray at-wavelength wavefront metrology at future high-brightness light sources. Our next steps include measuring strong focusing optical elements and exploring two-dimensional wavefront error, leveraging multiple movable edges and stitching algorithms to enhance measurement efficiency and precision.
